# Inhibition of CXCL12/CXCR4 axis as a potential targeted therapy of advanced gastric carcinoma

**DOI:** 10.1002/cam4.1085

**Published:** 2017-05-23

**Authors:** Li‐Jun Xue, Xiao‐Bei Mao, Li‐Li Ren, Xiao‐Yuan Chu

**Affiliations:** ^1^ Department of Medical OncologyJinling HospitalNanjing University Clinical School of MedicineNanjing210002China

**Keywords:** CXCL12, CXCR4, CXCR7, gastric carcinoma, inhibitor, targeted therapy

## Abstract

The whole outcome for patients with gastric carcinoma (GC) is very poor because most of them remain metastatic disease during survival even at diagnosis or after surgery. Despite many improvements in multiple strategies of chemotherapy, immunotherapy, and targeted therapy, exploration of novel alternative therapeutic targets is still warranted. Chemokine receptor 4 (CXCR4) and its chemokine ligand 12 (CXCL12) have been identified with significantly elevated levels in various malignancies including GC, which correlates with the survival, proliferation, angiogenesis, and metastasis of tumor cells. Increasing experimental evidence suggests an implication of inhibition of CXCL12/CXCR4 axis as a promising targeted therapy, although there are rare trials focused on the therapeutic efficacy of CXCR4 inhibitors in GC until recently. Therefore, it is reasonable to infer that specific antagonists or antibodies targeting CXCL12/CXCR4 axis alone or combined with chemotherapy will be effective and worthy of further translational studies as a potential treatment strategy in advanced GC.

## Introduction

Gastric carcinoma (GC) remains the top three most malignant tumors in global incidence rate during 2005–2015, with 1.3 million incident cases and 819,000 deaths worldwide in 2015 1. The majority of patients have metastatic disease at diagnosis or recurrent disease after resection, although surgery is currently the only curative treatment. Palliative chemotherapy has been shown to limitedly improve overall survival (OS) and quality of life for those with advanced GC; however, there are no universally accepted standard regimens until now 2.

Recent clinical evidence suggest that targeted drugs, trastuzumab and ramucirumab, significantly enhance chemotherapeutic efficacy and further extend the OS in advanced GC patients, through specifically inhibiting human epidermal growth factor receptor‐2 (EGFR‐2) and vascular endothelial growth factor receptor‐2 (VEGFR‐2), respectively 3, 4, 5. As for novel immune checkpoint therapies, ipilimumab and tremelimumab as anti‐cytotoxic T lymphocyte antigen‐4 (CTLA‐4) antibodies have got disappointing responses in phase 2 studies 6, 7. Pembrolizumab as anti‐programmed cell death protein‐1 (PD‐1) antibody is still under ongoing trials, although with encouraging partial response rates in phase 1 study 8. Despite these improvements, the prognosis of metastatic GC patients is very poor with median OS ranging from 4 to around 13 months, and thus exploration of alternative therapeutic targets is warranted 3, 9, 10.

Chemokine networks play important roles in the development and metastasis of various malignancies including GC, in which chemokine receptor 4 (CXCR4) and its chemokine ligand 12 (CXCL12) are two key factors in the cross‐talking between tumor cells and their microenvironment 11, 12. CXCL12 not only promotes the survival and proliferation of cancer cells via both autocrine and paracrine actions but also attracts the organ‐specific metastasis of CXCR4‐expressing tumors, which usually show more aggressive behaviors than those without CXCR4 expression 13, 14. CXCL12 and CXCR4 are always expressed at significantly increased levels in gastrointestinal cancers, which is associated with the activation of downstream pathways and survival, proliferation, angiogenesis, and migration of tumor cells 12. Furthermore, small molecular compounds, peptide antagonists, and specific antibodies against CXCR4 can efficiently inhibit downstream signaling of CXCL12/CXCR4 axis, block cancer dissemination and improve the outcome of patients 12, 15, 16.

Unlike many other solid tumors, there are still rare reported clinical trials about CXCR4 antagonists used in patients with GC until recently. However, more and more evidence indicate a promising implication of targeted therapy against CXCL12/CXCR4 axis in suppressing the growth and metastasis of GC. This review therefore aims to summarize and discuss multiple biological roles of CXCL12/CXCR4 axis and the potential application of CXCR4 inhibitors as a targeted therapy in advanced GC.

## Expression of CXCL12/CXCR4 Axis in GC

Both CXCL12 and CXCR4 have always been identified at significantly elevated levels in not only primary but also metastatic lesions of GC. Positivity rates for CXCL12 and CXCR4 at the primary cancer site reach 42.2–90.0% and 32.3–80.0% by immunohistochemistry (IHC) detection, respectively, which are significantly higher than those in the adjacent normal mucosa tissues 17, 18, 19, 20, 21, 22, 23, 24. In metastatic lymph nodes (LNs), positive staining rates of CXCL12 and CXCR4 are even as high as 66.7–94.4% and 91.7% by IHC, compared with those of 31.3–69.4% and 75% in the normal tissues, respectively 18, 20.

Expression profiles of CXCL12 and CXCR4 are closely related to biological behaviors of cancer cells and the outcome of patients with GC. CXCL12 expression is an independent prognostic factor for aggressive phenotypes of GC, including tumor size, invasion depth, lymphatic invasion and metastasis, TNM staging, surgical outcome, and the OS 22, 24, 25. The intensity of CXCR4 in primary GC lesion is positively associated with TNM staging, LN involvement, and recurrence/metastasis rate after radical surgery, but negatively with OS and disease‐free survival (DFS) 18, 26, 27, 28. As for the combination of CXCR4 and CXCL12 levels, CXCR4^high^/CXCL12^high^ in GC is significantly associated with tumor invasion depth, LN involvement, and higher TNM stage, which causes the worst prognosis, whereas patients with CXCR4^low^/CXCL12^low^ show the best prognosis 22. Some other studies indicate that only increased expression of CXCR4 but not CXCL12 is associated with the 5‐year survival and can be used as an independent prognostic biomarker, which may need further confirmation by more large‐sized prospective clinical trials 29.

There are still arguments about the relationships among CXCL12/CXCR4 expression, Lauren classification, and differentiation of GC. Some researchers found out that staining intensities of CXCR4 and CXCL12 were significantly higher and related to liver and LN metastases in intestinal‐type than in diffuse‐type GC 19, 30. However, He et al. reported that diffuse‐type GC presented higher CXCR4 level than intestinal‐type GC 27. Zhao et al. demonstrated that CXCR4 expression was related to poor differentiation of cancer cells, whereas Arigami et al. concluded that differentiated type of GC showed stronger CXCR4 expression than the undifferentiated type 20, 23.

In various human GC cell lines, CXCL12/CXCR4 axis exhibits different expression profiles. As reported, both CXCL12 and CXCR4 are highly expressed in MKN‐45, SGC‐790,1 and MKN‐28 cells and lowly or absently expressed in NUGC‐3, MKN‐1, and TMK‐1 cells, whereas high CXCR4 but low CXCL12 levels are detected in NUGC4, KATO III, AGS, and NKPS cells 17, 20, 25, 31, 32, 33. In addition, CXCR4 is also determined at high level in MGC‐803, HGC‐27, XN0422, MKN‐74, and SNU‐16 cells 20, 28, 31, 34. Notably, the major constitutive source of CXCL12 in solid tumors is factually stromal cells, including peritoneum mesothelial cells, vascular endothelial cells (VECs), and particularly cancer‐associated fibroblasts (CAFs) 17, 21. CAFs mediate integrin *β*1 clustering and promote invasion and metastasis of GC through the activation of CXCL12/CXCR4 signaling 25.

CXCL12 and CXCR4 are usually predominantly expressed on cell membrane or in the cytoplasm, although CXCL12 can also be secreted as a soluble form and CXCR4 sometimes still appears in the nucleus of GC cells 24, 28, 30, 35. In AGS cells, CXCR4 has been identified at high levels in both cytoplasm and nucleus, but low on cell membrane 35. KATO III cell line similarly expresses low level of membrane CXCR4, which shows specific migratory but no proliferative or anti‐apoptotic activities in response to CXCL12 28. As for MKN‐28, MKN‐45, MKN‐74, and SNU‐16 cells, CXCR4 is abundant in the cytoplasm but absent on cell membrane, which results in neither migratory, nor proliferative and survival responses to CXCL12 28. In primary GC tissues, CXCR4 is generally strongly expressed in both the cytoplasm and the nucleus 35. But in diffuse‐type GC, including signet‐ring cell carcinoma, CXCR4 staining is usually seen in the nucleus and scant in the cytoplasm 30. High level of nuclear CXCR4 tends to be correlated with poor differentiation, large tumor size, advanced stages, and short 5‐year OS in GC, which may be due to the fact that translocation of membrane CXCR4 into nucleus after binding to CXCL12 causes more invasive phenotypes 36.

## Roles of CXCL12 and CXCR4 in the Development and Metastasis of GC

**Figure 1:**
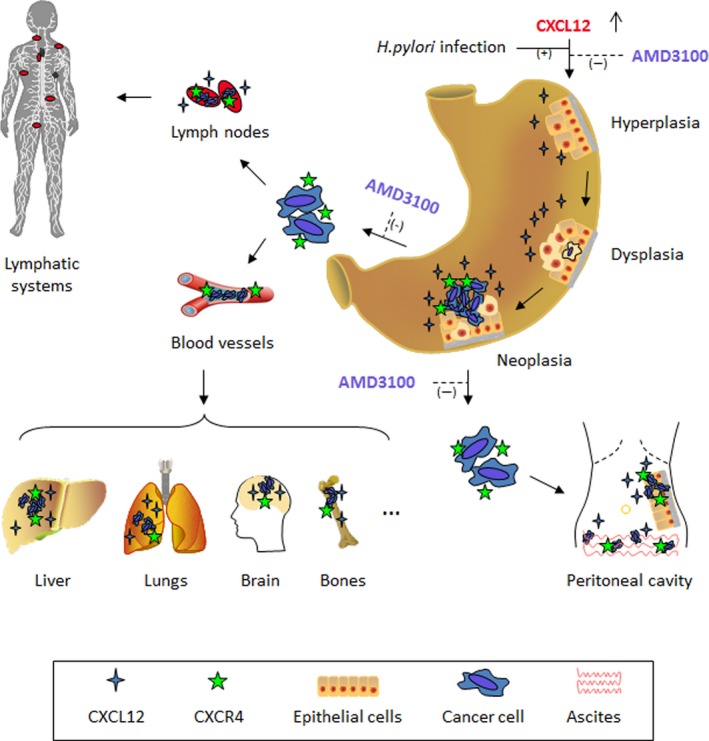
Roles of CXCL12/CXCR4 axis and its antagonist AMD3100 in the development and metastasis of gastric cancer.

Upregulation of CXCL12/CXCR4 axis contributes to significant gastric epithelial proliferation, hyperplasia, and dysplasia and promotes early carcinogenesis in a transgenic mouse model that expresses murine CXCL12 specifically in gastric parietal cells 37. In combination with inflammatory stimuli such as *Helicobacter* infection, overexpression of CXCL12 in stomach mucosa accelerates the process of spontaneous tumorigenesis (Fig. 1). Its carcinogenic mechanism is related with the CXCL12‐inducing recruitment of CXCR4‐positive CAFs and mesenchymal stem cells (MSCs) into tumor microenvironment (TME) 37. CXCL12 can also induce angiogenesis through activating the CXCR4 localized on VECs 38. The migration ability of VECs toward TME will be significantly increased under the stimulation of CXCL12 and inhibited by CXCR4 antagonist 39. Hence, CXCL12/CXCR4 axis should be a potential target not only for prevention of carcinogenesis, but for suppression of angiogenesis in GC 22, 37.

CXCL12/CXCR4 axis mediates the directional migration of CXCR4‐positive tumor cells to CXCL12‐expressing organs such as LNs and the liver 20, 40. It has been clinically and pathologically confirmed that CXCL12 and CXCR4 expressions are significantly associated with LN metastasis 41. CXCR4 is upregulated on lymphangiogenic endothelial cells (LECs) under the induction of VEGF‐C and mediation of hypoxia‐inducible factor‐1a (HIF‐1a), although its level is much lower in matured lymphatic vessels. CXCL12 as a chemoattractant stimulates lymphangiogenesis through CXCR4 by inducing the migration and tubule formation of LECs in an immunodeficient mouse model 42. In addition, CXCR4 expression is significantly associated with the selective metastasis of GC to liver 23, 30. Interestingly, normal hepatocytes mainly express CXCR4; but cancer cells in the metastatic liver express predominantly CXCL12 rather than CXCR4, which is opposite in the metastatic LNs 17, 30. Also, elevated CXCL12 level participates in the recruitment and homing of MSCs and CAFs into the TME of injured liver in immunocompetent animals, which helps promote hepatic metastases 37, 43.

CXCR4 positivity in primary lesions significantly correlates with the peritoneal metastasis of GC. And, CXCL12 is usually abundant in malignant ascites from patients with advanced GC 17. The peritoneum can attract CXCR4‐positive cancer cells to migrate toward and seed on through a CXCL12 gradient secreted by mesothelial cells 44. It is worth noting that Tsuboi et al. declared no significant correlations between CXCL12 and CXCR4 expressions with peritoneal metastasis or survival in pathological T3‐stage GC patients 21. However, their detection of free cancer cells in abdominal cavity might not be a reasonable evaluation method since intra‐abdominal‐free cancer cells may adhere to the peritoneum and then form colonized tumors by other mechanisms such as integrins and selectins 17, 21. Diffuse‐type GC cells may express higher CXCR4 than other types and tend to disseminate to the peritoneum 27. Fujita et al. have even identified CXCR4‐positive stem cells of diffuse‐type GC, which can penetrate gastric wall, migrate to CXCL12‐expressing peritoneum, and result in the formation of peritoneal tumor nodes and malignant ascites in an immunodeficient mouse model 45. Moreover, the formation of malignant ascites can be efficiently suppressed by antagonist of CXCR4 in immunodeficient mice engrafted with NUGC4 cells 17. Ding et al. reported that nude mice underwent intraperitoneal injection with both NUGC4 cells and CXCR4 antagonist, had fewer tumor numbers, and survived significantly longer than those only with cancer cells 46.

## Downstream Signaling Pathways of CXCL12/CXCR4 Axis in GC

The mitogen‐activated protein kinase (MAPK)/extracellular signal‐regulated kinase (ERK) and phosphoinositide 3‐kinase (PI3K) signaling are the two most pivotal downstream pathways of CXCL12/CXCR4 axis 40. CXCL12 recruits macrophages and myeloid cells and induces gastric epithelial proliferation through CXCR4 and its downstream ERK/PI3K pathways 37. In NUGC4 cells, CXCR4 mediates CXCL12‐induced rapid phosphorylation of ERK and Akt, which suppresses apoptotic signals of caspase‐9, caspase‐3, and Bcl‐2 and subsequently contributes to the proliferation and survival of GC 17. Upon CXCL12 stimulation, ERK 1/2 and Akt phosphorylation is also upregulated in LECs and essentially promotes the chemotactic cellular migration. Notably, the activation of ERK and Akt pathways by CXCL12 is independent of VEGF‐C/VEGFR‐3 signaling in enhancing the lymphangiogenesis 42. However, CXCL12 induces only the rapid phosphorylation of MAPK/ERK1/2 but not Akt in KATO III cells, which may indicate the variety and complexity of signaling in different GC cells although both NUGC4 and KATO III cell lines have similar features of signet‐ring cell carcinoma 28.

As the center of PI3K pathways, mammalian target of rapamycin (mTOR) regulates multiple processes including proliferation, survival, and migration of cancer cells. In MKN‐45 cells, CXCL12 induces the activation of PI3K/Akt/mTOR pathways through CXCR4 and subsequently stimulates cell migration by F‐actin reorganization and the activation of RhoA, Rac1, and Cdc42, which can be suppressed by the mTOR inhibitor rapamycin 32. Also, CXCL12 activates p70S6K and eukaryotic initiation factor 4E‐binding protein 1 (4E‐BP1) included in mTOR pathways which are located downstream of Akt in peritoneal disseminated GC 47. Moreover, rapamycin can inhibit the resulting enhanced metastatic properties such as matrix metalloproteinase (MMP) production, growth and migration of NUGC4 cells, and induce autophagic cell death, a type II programmed cell death 47.

## Antitumor Effects of CXCL12/CXCR4 Axis Inhibitors

Many chemical or biological inhibitors including small molecular compounds, peptides, and antibodies have been established with roles in suppressing the expression, binding, and downstream signaling of CXC12/CXCR4 axis 15, 16. Most of them are primarily used in the therapy of human immunodeficiency virus (HIV) infection or the mobilization and collection of CD34‐positive hematopoietic stem cells (HSCs) for transplantation in patients with non‐Hodgkin's lymphoma (NHL) or multiple myeloma (MM) 15, 48, 49. Recently, increasing evidence suggest that the blockage of CXCL12/CXCR4 axis still contributes to the antitumor effects in both hematologic and solid malignances.

In acute myeloid leukemia (AML), small molecule compounds AMD3100 and its analog AMD3465 are reported to enhance the efficacy of Ara‐C chemotherapy and extend the OS of immunocompetent mouse models 50, 51. According to a phase 1/2 study, AMD3100 significantly increases the remission rate and relapse‐free survival of combination chemotherapy (mitoxantrone, etoposide, and Ara‐C) in patients with relapsed AML 52. AMD3465 may increase the sensitivity of leukemic cells to Ara‐C through inhibition of CXCR4, which partially reduces the chemoprotection functions from stromal cells 51. Similarly, LY2510924 as a peptidic CXCR4 antagonist can rapidly and durably block surface CXCR4 and reverse stroma‐mediated chemoresistance of OCI‐AML3 cells to Ara‐C and doxorubicin. Rather than causing cell death, LY2510924 plays antileukemic roles mainly by suppressing the proliferation and progression of AML cells both in vitro and in an immunodeficient mouse model 53. Notably, small molecule antagonists including AMD3100 and AMD3465 show no antileukemia effects by monotherapy, although AMD3100 has got approval for clinical HSC mobilization as the first CXCR4 inhibitor 50, 51, 54, 55. Interestingly, LY2510924 alone can significantly prolong the survival of immunodeficient mice bearing OCI‐AML3 xenografts, which is further extended in combination with chemotherapy 53. Two other reports also demonstrate antitumor and apoptosis‐promoting effects of the peptide inhibitor TN140 and anti‐CXCR4 monoclonal antibody (mAb) MDX‐1338 as monotherapy in immunodeficient mouse models engrafted with AML, NHL, or MM cells, respectively 54, 55. TN140 appears to be more efficient than AMD3100 in regression of CXCR4‐expressing AML cells and significantly prolongs the survival of xenografted immunodeficient mice 54. BKT140, a high‐affinity CXCR4 antagonist, can even effectively inhibit those NHL cells hidden in the bone marrow of an immunodeficient mouse model, directly cause apoptotic cell death and reduce the stroma‐induced rituximab resistance 56. When used in combination with rituximab, BKT140 synergistically enhances the cytotoxic antilymphoma effects through inducing mitochondrial damage, caspase‐3‐associated apoptosis, and reversion of the G2–M arrest 56.

CXCR4 inhibitors still show antitumor effects in many solid malignancies (Table 1). AMD3100 significantly suppresses the growth of human anaplastic thyroid cancer cells, sphere‐forming activity of various non‐small cell lung cancer (NSCLC) cell lines, perineural invasion of adenoid cystic carcinoma, and the angiogenesis and lung metastasis of chodrosarcoma cells 57, 58, 59, 60. AMD3100 also inhibits the growth of intracranial glioblastoma and medulloblastoma xenografts in immunodeficient mice by increasing apoptosis and decreasing the proliferation of tumor cells 61. Similarly, AMD3465 significantly reduces the growth of medulloblastoma and glioblastoma cells both in vitro and in nude mouse models through the down‐regulation of cyclic AMP 62. AMD3465 also suppresses tumor formation, invasiveness, and metastases to the lung and liver in murine syngeneic immunocompetent breast cancer models 63. LY2510924 exhibits drastic inhibition of both tumor growth of kidney, lung, and colon cancer cells, and lung metastases of breast cancer cells in corresponding immunodeficient mouse models 64. According to a phase I trial, LY2510924 is clinically safe and well tolerated in advanced solid cancers variously originated from colorectum, lung, breast, pancreas, prostate, and other organs, with primary response being 20% of stable disease (SD) 65. MSX‐122, another small molecule compound, is shown to effectively decrease both liver micrometastases of melanoma and lung metastases of breast cancer and squamous cell carcinoma of the head and neck (SCCHN) in nude mouse models 66. Notably, besides increasing tumor apoptosis and necrosis, AMD3100 still promotes antitumor T‐cell responses by greatly reducing the infiltration of intratumoral FoxP3 + regulatory T (Treg) cells, which contributes to a significant OS advantage in an immunocompetent mouse model of ovarian cancer 67.

**Table 1:** Studies of antitumor effects of CXCR4 inhibitors used in solid malignancies

Cancer type	CXCR4 inhibitor	Author (Year)	Reference
Adenoid cystic carcinoma	AMD3100	Jeong et al. (2014)	60
Breast cancer	AMD3465	Ling et al. (2013)	63
	CTCE‐9908	Hassan et al. (2011)	77
		Huang et al. (2009)	80
		Richert et al. (2009)	81
	LY2510924	Peng et al. (2015)	64
		Galsky et al. (2014)	65
	MSX‐122	Liang et al. (2012)	66
	Nef	Bumpers et al. (2013)	71
	TN14003	Liang et al. (2004)	72
Chondrosarcoma	AMD3100	Sun et al. (2013)	59
Colorectal carcinoma	LY2510924	Peng et al. (2015)	64
		Galsky et al. (2014)	65
	Nef	Bumpers et al. (2005)	70
Esophageal cancer	CTCE‐9908	Drenckhan et al. (2013)	79
Gastric cancer	AMD3100	Izumi et al. (2016)	25
		Xie et al. (2010)	122
		Yasumoto et al. (2006)	17
Glioblastoma and medulloblastoma	AMD3100	Rubin et al. (2003)	61
	AMD3465	Yang et al. (2007)	62
Melanoma	AMD3100	Portella et al. (2013)	69
	CTCE‐9908	Kim et al. (2008)	78
	MSX‐122	Liang et al. (2012)	66
	R, S and I peptides	Portella et al. (2013)	69
Non‐small cell lung cancer	AMD3100	Jung et al. (2013)	58
	BKT140	Fahham et al. (2012)	68
	LY2510924	Peng et al. (2015)	64
Osteosarcoma	CTCE‐9908	Kim et al. (2008)	78
	R, S and I peptides	Portella et al. (2013)	69
Ovarian cancer	AMD3100	Righi et al. (2011)	67
	CTCE‐9908	Kwong et al. (2009)	82
Pancreatic cancer	LY2510924	Galsky et al. (2014)	65
	TN14003	Mori et al. (2004)	74
Prostate cancer	CTCE‐9908	Porvasnik et al. (2009)	76
	LY2510924	Galsky et al. (2014)	65
Renal cell carcinoma	AMD3100	Portella et al. (2013)	69
	LY2510924	Peng et al. (2015)	64
	R, S and I peptides	Portella et al. (2013)	69
Small cell lung cancer	TN14003	Hartmann et al. (2005)	75
Squamous cell carcinoma of the head and neck	MSX‐122	Liang et al. (2012)	66
	TN14003	Yoon et al. (2007)	73
Thyroid cancer	AMD3100	De Falco et al. (2007)	57

Therapeutic effects of peptide CXCR4 antagonists have also been verified in various solid cancer models. In human NSCLC, BKT140 as a small peptide not only significantly delays tumor development in xenografted nude mice, but reduces the proliferation capacity of cancer cells and augments both efficacies of chemotherapy (cisplatin or paclitaxel) and radiotherapy in vitro 68. R, S, and I peptides dramatically inhibit the growth of human renal cancer cells in nude mice and lung metastases of murine melanoma and osteosarcoma xenografts in immunocompetent mice 69. Nef protein and related peptides are found to significantly enhance the apoptosis and suppress the growth of human colon and breast cancer cells 70, 71. TN14003 is reported to effectively inhibit the proliferation, invasion, and migration of human pancreatic cancer cells in vitro, and significantly limit primary tumor growth and block lung metastases of both human SCCHN and breast cancer xenografts in immunodeficient mouse models 72, 73, 74. TN14003 still antagonizes the protection of SCLC cells from etoposide‐induced apoptosis by CXCL12‐expressing stromal cells 75. In addition, CTCE‐9908 significantly inhibits the proliferation of human prostate cancer cells and reduces tumor size of xenografts in immunodeficient animals 76. CTCE‐9908 suppresses not only the proliferation and migration in vitro, but organic metastases of osteosarcoma and melanoma xenografts in immunocompetent mice, and esophageal and breast cancer cells in immunodeficient models, respectively 77, 78, 79, 80. However, CTCE‐9908 is shown to decrease metastatic burden but not incidence of metastasis in the major organs (lungs, bone, heart, liver, kidneys, pancreas, and spleen), most significantly in the bone 81. In ovarian cancer cells, CTCE‐9908 alone induces multinucleation, G2‐M arrest, and abnormal mitosis, and leads to an additive cytotoxicity when combined with paclitaxel chemotherapy 82. Furthermore, antitumor and anti‐metastatic effects of chemotherapy (docetaxel) and anti‐VEGFR2 therapy (mAb DC101) are markedly enhanced by CTCE‐9908 in a mouse mammary tumor virus (MMTV)‐driven Polyoma Middle T Antigen (PyMT) transgenic mouse model of breast cancer with HER2/neu overexpression, suggesting a potential novel strategy of combined therapies against cancer 77.

## Cross‐Talks of CXCL12/CXCR4 Axis with Other Chemokines and Chemokine Receptors

CXCL12/CXCR4 axis has complex cross‐talks with some other chemokines and chemokine receptors. CXCR7, initially named receptor dog cDNA 1 (RDC1), can bind not only to CXCL12 with higher affinity than CXCR4 but to CXCL11, which still acts as one of the ligands for CXCR3 besides CXCL10 and CXCL9 83, 84. The binding of CXCR4 to CXCL12 is directly competed by CXCR7, and at the same time indirectly influenced by the interactions among CXCR3 and CXCL9, CXCL10 and CXCL11.

CXCR7 is usually upregulated in GC tissues with a positivity rate of 63–84.62% by IHC, which correlates with deep invasion, LN metastasis, advanced stages, and bad outcome of patients 85, 86, 87, 88, 89. Also, CXCR7 is highly expressed in the majority of tumor‐associated vessels and related with tumor neovascularization via regulating the secretion of proangiogenic factors such as interleukin‐8 and VEGF 90, 91, 92. CXCR7 overexpression may affect disease progression by stimulating proliferation, invasion, migration, adhesion, and angiogenesis of GC through *β*‐arrestin‐dependent downstream signalings, including Akt, ERK1/2, p38 MAPK, JAK2/STAT3, and stress‐activated protein kinase (SAPK) pathways 91, 93, 94, 95, 96. CXCL11, also known as interferon‐inducible T‐cell *α* chemoattractant (I‐TAC), induces chemoattraction and activation of T lymphocytes through CXCR3 that is preferentially expressed on both polarized type 1 helper T (Th1) and cytotoxic T lymphocytes 97, 98, 99. In GC lesions, lower level of CXCL11 generally suggests a special immune dampening in the TME and is independently associated with a shorter OS 98. CXCR3 is overexpressed in GC tissues and cell lines, which significantly correlates with the survival of patients although with directional arguments 100, 101, 102. Zhou et al. reported that high level of CXCL10/CXCR3 axis activated the PI3K/Akt pathway‐dependent MMP‐2 and MMP‐9 productions, which caused vascular invasion, LN involvement, later stages, and poor prognosis 100. Nevertheless, some other researchers concluded that overexpression of CXCR3 as an independent favorable prognostic factor for the OS was inversely associated with invasion depth and metastatic status of GC 101, 102. The mechanism may be attributed to increased infiltrations of CD4^+^ and CD8^+^ tumor‐infiltrating lymphocytes, which is supported by the findings that upregulation of both CXCR3 and its chemokine ligands results in enhanced antitumor responses of T cells 97, 99, 101. In addition, CXCR3 can form at least two splice variants including CXCR3‐A predominantly expressed in epithelial cells and CXCR3‐B, which is expressed on fibroblasts and endothelial and epithelial cells. Both CXCR3‐A and CXCR3‐B are shown to interact with CXCL11, CXCL10, and CXCL9, leading to reciprocal roles of pro‐ and antitumorigenesis, respectively, through activating proliferative or inhibitory signals via two different G proteins (CXCR3‐A‐Gi/CXCR3‐B‐Gs) 92, 103, 104, 105.

CXCR7 binds to CXCL12 with about 10 times higher affinity than CXCR4 (Kd = 0.4 nmol/L vs. 3.6 nmol/L), and with 10‐ to 20‐fold greater affinity than to CXCL11 84, 94, 106. CXCL12 exists as dimers or is secreted mainly as monomers, in which CXCR7 preferentially interacts with the latter 95, 107, 108. Dimeric CXCL12 induces downstream calcium flux but not chemotaxis, which may depend on the cross‐talk between CXCR7 and monomeric CXCL12 107, 109. Furthermore, CXCR7 and CXCR4 can form homo‐ and heterodimers when coexpressed in vivo or in vitro, which has been confirmed by their presence on HEK293 cells mediated by CXCL12 110, 111. CXCR7/CXCR4 heterodimers constitutively recruit *β*‐arrestin and play important roles in the modulation of a broader panel of downstream pathways, such as delaying ERK1/2 activation, enhancing calcium flux and MAPKp42/44 phosphorylation, and decreasing the CXCR4‐mediated Gi activation and calcium signaling 83, 84, 95, 96, 110, 111, 112, 113, 114. Notably, CXCR7 as a scavenger receptor with molecular sink activity still can modulate or demarcate both CXCL12 and CXCL11 gradients in biological processes of inflammation and cellular infiltration and migration 115, 116. Binding of CXCL12 to CXCR7 leads to their internalization to the cytoplasm, where CXCL12 will be transported to lysosomes for degradation and then CXCR7 recycles back to cell membrane 106, 117. CXCR7 may also pull CXCR4 when it has bound CXCL11 or vice versa with different CXCR4 ligands 95. The internalization and sorting of CXCR4 to lysosomes mainly depends on the ubiquitination of its lysine residues upon ligand binding 118. As reported, coexpression of CXCR7 and CXCR4 not only synergistically enhances *β*‐arrestin‐dependent invasion and migration via activating ERK1/2 MAPK pathway, but also inhibits CXCR4‐induced PI3K/MAPK metastatic signals by CXCR7‐mediated sinking of monomeric CXCL12, which are factually opposite functional capabilities 96, 119. Moreover, the blocking of CXCR7 by antagonists cannot change CXCR4‐mediated Akt and ERK phosphorylation in those cells with both the expression of intracellular CXCR7 and membrane CXCR4, which suggests that CXCR7 should not be necessary for CXCR4‐mediated signaling 120. Relationships among CXCR4, CXCR7, CXCL12, and CXCL11 in the context of cancer behaviors are always highly dynamic, since tissue concentrations of these receptors and chemokines are being regulated by many factors such as hypoxia 92, 93.

It is demonstrated that inhibition of CXCR7 pathway leads to an antitumor activity in various solid malignancies originated from colon, liver, pancreas, and head and neck 90. CCX754 as one of CXCR7 antagonists significantly suppresses the growth of lung cancer in both immunodeficient and immunocompetent mouse models, which are engrafted by human A549 and mouse LL/2 Lewis lung carcinoma cell lines, respectively 94. Notably, AMD3100, the first clinically used CXCR4 inhibitor, can also bind to CXCR7, but acting as an allosteric agonist with distinct effects in promoting CXCL12 binding and the potency of CXCL12‐induced *β*‐arrestin recruitment to CXCR7 83, 121. The maximal effect of CXCL12 on the CXCR7 homodimer conformation is also increased by 40% by AMD3100, which may indicate an AMD3100‐mediated activation of CXCR7 pathways 116. However, there are still rare reports about the efficacy of specific inhibitors of CXCR7 including CCX754, CCX771, CCX733, and CCX2066 in GC until now 90.

## Potentials and Limitations of Targeted Therapy Against CXCL12/CXCR4 Axis in GC

The block of CXCL12/CXCR4 axis leads to similar antitumor effects in GC like in other malignancies. As reported, anti‐CXCR4 antibody significantly suppresses the proliferation of NUGC4 cells induced by CXCL12 through its neutralizing role 17. AMD3100 is efficient at inhibiting the proliferation, invasion, and migration of GC cells by attenuating not only the downstream signaling of CXCL12/CXCR4 axis but also interactions between TME and cancer cells 25. Furthermore, AMD3100 effectively enhances the docetaxel chemosensitivity in GC through inhibitions of CXCR4 expression and downstream pathways 122. In a xenografted nude mouse model of NUGC4 cells, AMD3100 is confirmed to reduce both peritoneal carcinomatosis and malignant ascites formation, which indicates novel therapeutic implications of CXCR4 antagonists in peritoneal metastasis of GC 17.

Up to now, there have been rare studies or trials focused on the therapeutic efficacy of CXCR4 inhibitors in GC 16, 17. However, it is worth mentioning that there are similar findings about the roles of CXCL12/CXCR4 axis and its inhibitors in GC as in many other solid carcinomas. First, the upregulation of CXCL12/CXCR4 axis and activation of downstream pathways and their biological functions in proliferation, invasion, and migration of cancer cells are similar. Second, TME‐dependent conditions such as hypoxia are similar. Third, the effectiveness of CXCR4 antagonists in blocking the expression and downstream signaling of this axis is similar. Fourth, the roles of CXCR4 antagonists in suppressing tumor growth, invasion, and metastasis are similar both in vitro and in vivo. Fifth, CXCR4 inhibitors can similarly enhance the sensitivity of cancer cells to certain chemo drugs. Therefore, it is reasonable to infer that specific antagonists or antibodies targeting CXCL12/CXCR4 axis alone or combined with chemotherapy should be effective and worthy of further studies as a potential treatment strategy in advanced GC, just like in other malignancies.

However, certain limitations of the targeting strategy against CXCL12/CXCR4 axis should be noted. Firstly, side effects of CXCL12/CXCR4 axis inhibitors upon extra‐tumor tissues or cells exist objectively, although some inhibitors have been clinically approved. Functions and status of normal epithelial cells, immune cells, lymphatic vessels, angiogenesis, and hematopoiesis might need to be especially focused on 11, 84. Also, allosteric effects of specific molecular inhibitors may interfere with their anticipated efficacy upon primary receptors 83, 121. For example, AMD3100 can bind to and activate CXCR7 and bind to but inhibit CXCR4. In addition, therapeutic effects of these axis inhibitors may be influenced by other cross‐talks among several chemokines and receptors, including mainly CXCL12‐CXCR7, CXCL11‐CXCR7, and even CXCL11/CXCL10/CXCL9‐CXCR3 interactions 83, 84. Considering the prominent role of CXCR7 competition with CXCR4 for binding to CXCL12, blockage of CXCR4 probably only partially inhibits the responsiveness of cancer cells to CXCL12 gradient 83. It is thus speculated that blocking both CXCR4 and CXCR7 receptors could be more efficient in the inhibition of biological effects of CXCL12, than just suppressing one of them 123. Nevertheless, the loss of CXCR7 expression during mouse embryogenesis coincides with the lethal consequence of CXCR4 or CXCL12 genetic deletion, which might suggest a special caution in the combination of various inhibitors 94. Detailed researches on the functions of CXCL12‐CXCR4/CXCR7 pathways and their cross‐talks with CXCR3, CXCL11, CXCL10, and CXCL9 remain urgently warranted, which help lead to safer and more efficient use of their molecular inhibitors in targeted cancer therapy.

## Conflict of Interest

The authors have no conflict of interest to disclose.
